# Purinosomes involved in the regulation of tumor metabolism: current progress and potential application targets

**DOI:** 10.3389/fonc.2024.1333822

**Published:** 2024-04-26

**Authors:** Jiaqi Xie, Jiaqi Liu, Xiehui Chen, Changchun Zeng

**Affiliations:** ^1^ Institute for Brain Research and Rehabilitation, South China Normal University, Guangzhou, China; ^2^ Department of General Medicine, Shenzhen Longhua District Central Hospital, Shenzhen, China; ^3^ Xianning Medical College, Hubei University of Science and Technology, Xianning, China; ^4^ Department of Geriatric Medicine, Shenzhen Longhua District Central Hospital, Shenzhen, China

**Keywords:** purinosome, *De novo* purine synthesis, tumor metabolism, AMPK signaling pathway, mTOR signaling pathway, HIF-1 signaling pathway

## Abstract

The core of tumor cell metabolism is the management of energy metabolism due to the extremely high energy requirements of tumor cells. The purine nucleotide synthesis pathway in cells uses the purinosomes as an essential spatial structural complex. In addition to serving a crucial regulatory role in the emergence and growth of tumors, it contributes to the synthesis and metabolism of purine nucleotides. The significance of purine metabolism in tumor cells is initially addressed in this current article. The role of purinosomes as prospective therapeutic targets is then reviewed, along with a list of the signaling pathways that play in the regulation of tumor metabolism. A thorough comprehension of the function of purinosomes in the control of tumor metabolism can generate fresh suggestions for the creation of innovative cancer treatment methods.

## Introduction

### Metabolic regulation of tumor cells

Tumor cells have different metabolic properties than normal cells, the most notable of which is a high reliance on nutrients, particularly glucose and glutamine. Increased glucose absorption is a typical feature in tumor cells. The Warburg effect exists in tumor cells and is shown by enhanced glycolysis and lactic acid production, which are hallmarks of tumor cell metabolism (1). Many tumor cells are glutamine-dependent, because glutamine provides cells with the carbon and nitrogen they require for biosynthesis (2). Glutamine is a significant mitochondrial substrate and glutamine catabolism gives cells a potent source of NADPH synthesis. In addition, tumor cells are able to utilize other metabolic pathways for energy, such as fatty acid oxidation and amino acid metabolism (3, 4).

Tumor cells require constant energy and biomolecule acquisition to support their aberrant proliferative activity during growth and reproduction. Purines are a type of biomolecule that is primarily utilized to construct nucleic acids and energy transfer molecules (such as ATP and GTP). Purine metabolism in tumor cells has a unique requirement for their rapid growth and proliferation. Tumor cells often oversynthesize purines to meet their rapid proliferation needs, and they also supply the precursor molecules such as glucose-6-phosphate and phosphoribosyl to produce purines through the glycolytic pathway. As a result of their high metabolic demands, tumor cells may modify purine metabolic pathways to support their growth, division, and proliferation. For example, tumor cells directly regulate several genes encoding key enzymes in the purine biosynthesis pathway, such as ADSL and IMPDH2, through the transcription factor MYC, and then up-regulate enzymes related to purine synthesis to maintain cell self-renewal and proliferation (5–7). Thus, purine metabolic pathways in tumor cells can be studied to learn more about how to design treatment approaches that specifically target cancer cells.

Tumor metabolic regulation not only affects the metabolic state of tumor cells themselves, but also affects the activation of immune cells, the generation of tumor-related blood vessels, as well as the invasion and metastasis of tumor cells by regulating the metabolic state of tumor microenvironment (8). Lactate acidification is a metabolic characteristic found in tumor tissues. Purine metabolism in tumor cells can influence lactate generation and acidification processes. Purine metabolism disorders may result in lower intracellular ATP levels, which activate the acidification pathway and allow tumor cells to adapt to the malignant environment by lactate acidification. Because of the accumulation of the glycolytic metabolite lactate, it can lower the pH value in the microenvironment and provide a favorable survival environment for tumor cells, which promotes tumor growth, invasion, and development (9, 10) as well as immune escape (11).

The management of tumor metabolism is critical for energy supply, biosynthesis, tumor cell growth and transformation, and tumor microenvironment regulation. An in-depth understanding of tumor metabolic regulatory mechanisms is extremely valuable for developing new cancer therapy techniques and improving treatment outcomes.

Beginning with an overview of the significance of purine metabolism in tumor cells, this paper goes on to discuss the identification of purinosomess and the current state of research into their composition and roles. Next, in an effort to generate fresh concepts for the creation of novel tumor treatment approaches, we enumerated the signaling pathways associated with purinosomes in the control of tumor metabolism, including the AMPK, mTOR, and HIF-1 signaling pathways. We also talked about the potential of purinosomes as therapeutic targets.

## Discovery and function of purinosome

*De novo* purine synthesis is a ten-step process that produces inosine 5’ -monophosphate (IMP), adenosine 5’ -monophosphate (AMP), and guanosine 5’ -monophosphate (GMP). It is catalyzed by six different enzymes and can be split into two stages: the synthesis of IMP comes first, and then AMP and GMP are produced. Two phases come together to form the IMP. Three monofunctional enzymes phosphoribosyl pyrophosphate amidotransferase (PPAT), formylglycinamidine ribonucleotide synthase (FGAMS), and adenylosuccinate lyase (ADSL), two bifunctional enzymes phosphoribosyl aminoimidazole succinocarboxamide synthetase (PAICS) and IMP cyclohydrolase (ATIC), and a trifunctional enzyme GARS-GAR transformylase-aminoimidazole ribonucleotide synthetase (TrifGART) are among the enzymes involved in *de novo* purine synthesis. The *de novo* purine synthesis route involves several different enzymes, each of which is crucial to the process. Thus, some scientists started to question whether there was anything that organisms could do to ensure that this chain of events occurred frequently enough to guarantee the effective production of purines for cells.

Back in 2008, Songon An et al. published an article in *Science* on reversible compartmentalization of *de novo* purine biosynthesis complexes, which first proposed the concept of purinosomes. Purinosomes are punctate multienzyme complexes that are reversibly assembled by enzymes in the *de novo* purine synthesis pathway (12). Hong Zhao et al. found higher levels of IMP in cells rich in purinosomes when comparing cells treated with 2-dimethylamino-4,5,6,7-tetrabromo-1H-benzimidazole (DMAT), a casein kinase 2 (CK2) inhibitor that induces the development of purinosomes, to cells cultivated in purine removal media. Furthermore, when cells were cultivated under purine depletion conditions, *de novo* manufacture of IMP rose by 50%. Purinosomes, it is determined, are functional compounds that increase *de novo* metabolic flux when purine demand is high (13). Minjoung Kyoung et al. promptly inserted GFP-labeled purine remedy production pathway enzyme human hypoxanthine phosphoribosyltransferase 1 (HPRT1) into Hs578T cells, but under purine depletion circumstances, HPRT1-GFP did not create purinosomes in Hs578T cells. HPRT1-GFP co-transfection with FGAMS-OFP did not form purinosomes in purine-depleted Hs578T cells. Under purine depletion conditions, the human purine remedy production route is shown to be independent of purinosome assembly (14).

Purinosomes are now characterized using a range of biological approaches. Anthony M. Pedley and Stephen J. Benkovic developed a method to visualize purinosomes in living cells and a method to detect purinosome in fixed cells with the help of immunofluorescence (15). Meanwhile, In addition to the immunofluorescence technique, the study by Chung Yu Chan et al. also referred to the basic morphological characterization, that is, the average size and average quantity of purinosomes in a cell were used as the physical criteria to distinguish purinosomes from other cell bodies (16). Yijun Deng et al. probed protein-protein interactions within purinosomes with a novel application of the Tango PPI reporter system and found that three enzymes in the *de novo* purine biosynthesis pathway, PPAT, TrifGART, and FGAMS, form the core of purinosomes (17). Based on diffusion coefficient data obtained for six *de novo* purine biosynthesis enzymes in purine depleted medium, Minjoung Kyoung et al. discovered that PPAT, TrifGART, and FGAMS had similar diffusion coefficients, and PAICS and ADSL had similar diffusion coefficients. Three intermediates are proposed for enzymes involved in *de novo* purine biosynthesis in cells during purine deficiency (14).

Some investigations have discovered that in the presence of purine deficit, treating cells with nocodazole, a small molecule inhibitor of microtubule polymerization, causes microtubule depolymerization, which is not beneficial to the creation of purinosomes. Even if the medium is poor in purine, the amount of *de novo* purine biosynthesis by cells is reduced. Purinosomes were clearly integrated in microtubule networks, according to cellular imaging of the cytoskeleton structure in the presence of purinosomes. In conclusion, a microtubule network organizes the spatial distribution of purinosomes, and tubulin disintegration inhibits *de novo* purine biosynthesis (18).

*De novo* purine biosynthesis necessitates ATP consumption, and scientists are intrigued by the existence of correlations in the subcellular localization between purinosomes and mitochondria. Jarrod B. French et al. used 3D random optical reconstruction microscopy to show that purinosomes colocalize with mitochondria by imaging HeLa cells under conditions that encourage purinosomes production (19). Julie Williamson et al. found that under normal physiological conditions purine biosynthetic enzymes are present in all compartments of hippocampal neurons and that these enzymes are localized around mitochondria (20). Colleen A. Mangold et al. used HSV-1 virus infection as a natural cellular stressor to initiate high purine demand, discovering that *de novo* purine biosynthesis may occur at specific local sites within neurons and that this phenotype is conserved across species. Colocalization of FGAMS with mitochondria was also identified in neurons, implying that the functional relationship between mitochondria and *de novo* purine biosynthesis is conserved across cell types (21).

Vidhi Pareek et al. proved the existence of purinosomes directly in 2020 by using metabolomics and gas cluster ion beam secondary ion mass spectrometry to perform *in situ* three-via microchemical imaging of single cells to directly visualize multienzyme complexes of purinosomes that act synergistically to increase pathway flux by approximately 7-fold (22).

## Characteristics of purinosome

### Cell cycle dependence

Purinosomes self-assemble in response to the cell’s purine requirements. Purinosomes assemble and deconstruct themselves during the phase of cell division because it has the largest need for purines. This is known as cell cycle dependence. Studies demonstrated that the amount of purinosomes in Hela cells rises during the G1 phase, when the cells prepare the material for DNA replication (16). The availability of phosphoribosyl pyrophosphate (PRPP) and Pi affects purine synthesis that is reliant on the cell cycle. Since PRPP is the rate-limiting substrate in the first step of the *de novo* purine biosynthesis pathway and PRPP synthetase activity increases between G1 and S phases, purine synthesis increases when cells move from G1 to S phase (23).

### Reversibility of assembly

Purinosome construction is reversible, allowing it to be carried out in response to the need for intracellular purine. A research shows that the proportion of purinosome-positive cells in Hela cells peaked in the G1 phase and gradually declined in the S and G2/M phases (16). Due to their purine-deficient development environment, the G1 phase cells have the highest purine demand, which stimulates the *de novo* purine biosynthesis pathway and produces a large amount of purinosomes. However, as cell growth continues, the need for purine gradually declines. Because the purine metabolites already present in the cell might activate the rescue purine synthesis pathway, this results in a reduction in the number of positive cells. After 12 hours, there were less positive cells because there were fewer cells in the G1 phase. This offers more proof that purinosome assembly is reversible and that it may be dynamically modified to meet the needs of the cell (16). It has been found that sequential addition of two CK2 inhibitors, DMAT and 4,5,6,7-tetrabromobenzotriazole (TBB), to HeLa cells can reverse purinosomes assembly. This finding directly demonstrated the reversibility of purinosomes (24, 25).

## Mechanism of purinosome in the regulation of tumor metabolism

### AMPK signaling pathway

AMP-activated protein kinase (AMPK) is an important cellular energy-sensing protein kinase and a key regulator of purine synthesis. It directly regulates nucleic acid synthesis in tumor cells (26). The role of AMPK as the cell’s primary metabolic hub, which is activated whenever the AMP/ATP ratio is high, has been found to suppress the growth of a number of cancer cells as well as the mammalian target of rapamycin (mTOR) signaling pathway. The metabolite 5-aminoimidazole-4-carboxamide ribonucleotide (AICAR) is an allosteric activator of AMPK and the substrate of the last enzyme in the *de novo* purine synthesis pathway. Increased *de novo* synthesis pathways of purine nucleotides often imply increased assembly of purinosomes, which will lead to the accumulation of AICAR, and increased endogenous AICAR activates AMPK and its downstream signaling pathways (27–29).

**Figure 1:**
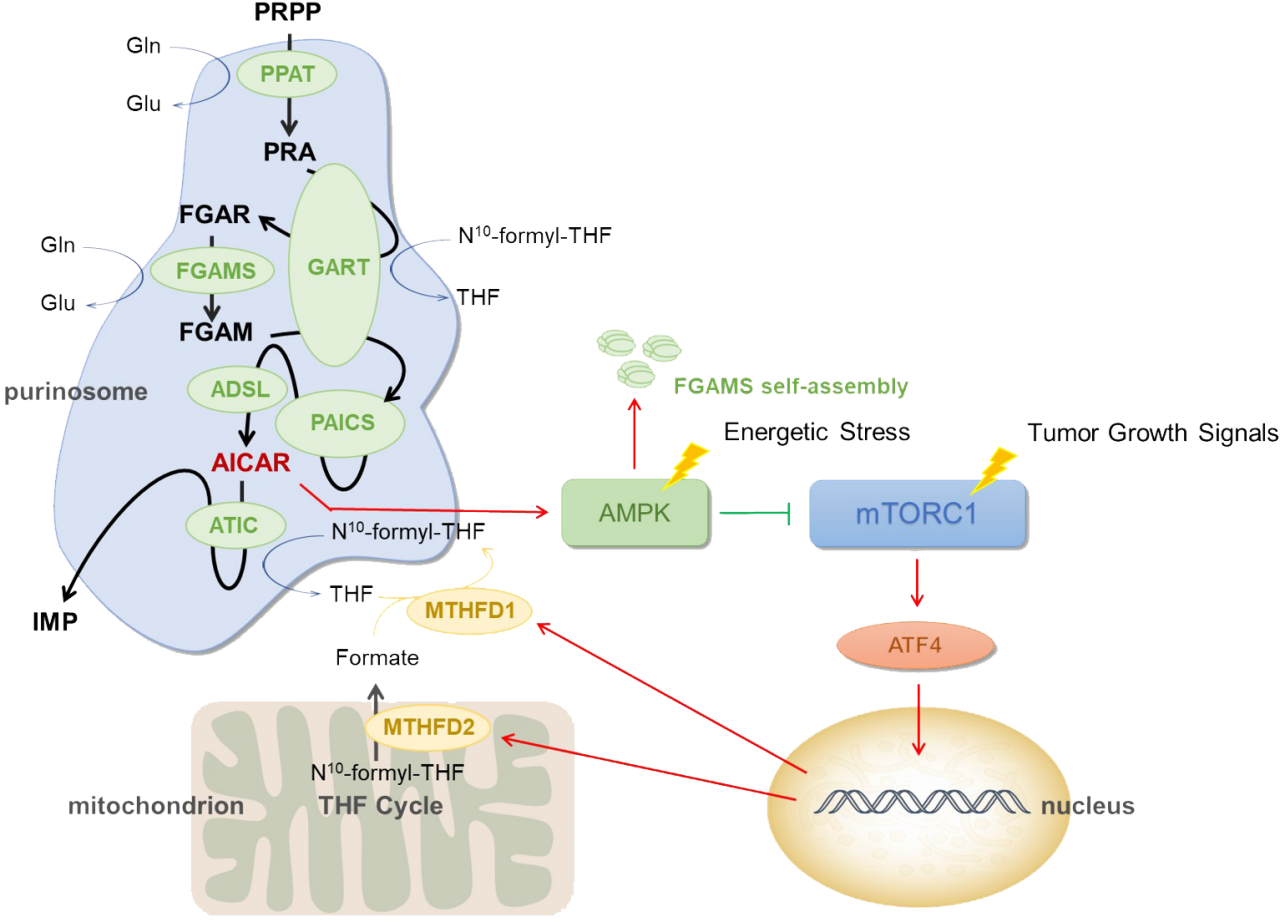
Mechanism of purinosome. Low blood sugar, hypoxia, ischemia, and other conditions are examples of energy stress. When cells are subjected to these energy stresses, they may cause purinosomes assembly and increase de novo purine synthesis. PRPP, phosphoribosyl pyrophosphate; PPAT, phosphoribosyl pyrophosphate amidotransferase; PRA, phosphoribosyl amine; GART, GARS-GAR transformylase-aminoimidazole ribonucleotide synthetase; FGAR, formylglycineamide ribotide; FGAMS, formylglycinamidine ribonucleotide synthase; FGAM, formylglycineamidine ribotide; PAICS, phosphoribosyl aminoimidazole succinocarboxamide synthetase; ADSL, adenylosuccinate lyase; AICAR, 5-aminoimidazole-4-carboxamide ribonucleotide; ATIC, IMP cyclohydrolase; IMP, inosine 5’ -monophosphate; N10 -formyl-THF, N10 -formyl tetrahydrofolate; THF, tetrahydrofolate; Gln, glutamine; Glu, glutamate; MTHFD1, methylenetetrahydrofolate dehydrogenase 1; MTHFD2, methylenetetrahydrofolate dehydrogenase 2; AMPK, AMP-activated protein kinase; mTORC1, mammalian target of rapamycin complex 1.

Based on the information provided earlier, purinosomes are made up of a number of enzymes involved in *de novo* purine synthesis, each of which is essential and very important. The research demonstrated that after treatment with an AMPK activator, FGAMS, one of the six pathway enzymes involved in *de novo* purine synthesis, exhibited single-enzyme self-assembly. Lower levels of purine metabolites such as IMP, AMP, GMP, and ATP in HeLa cells show a downregulation of *de novo* purine synthesis caused by AMPK’s encouragement of FGAMS self-assembly. The study revealed that the downregulation of purine production in human cells is driven by AMPK’s spatial isolation of FGAMS, and indirectly demonstrated that AMPK signaling pathway has a certain influence on purinosomes assembly (30). The actual pathway can be referred to **Figure 1**. Furthermore, it has been discovered that by raising the quantity of AMP within cells, ADSL activation might lead to AMPK activation (31). Additionally, it has been demonstrated that AICAR buildup brought on by ATIC enzyme inhibition might indirectly activate AMPK (32, 33). When considered collectively, these findings point to AMPK’s significant regulatory function in the *de novo* purine synthesis.

### mTOR signaling pathway

Mammalian target of rapamycin is a key protein that controls cell proliferation and metabolism, and overactivation of this protein has been linked to the incidence and growth of numerous cancers. By controlling the mitochondrial tetrahydrofolate cycle, mTOR influences how purinosomes are produced and disassembled (34). mTORC1 signaling is connected to the expression of the mitochondrial enzyme methylenetetrahydrofolate dehydrogenase 2 (MTHFD2) in both healthy and malignant cells. Activating transcription factor 4 (ATF4) stimulates the gene expression and serine biosynthesis necessary for the mitochondrial tetrahydrofolate (mTHF) pathway to produce formate (35). In order to increase the generation of formyl units needed for *de novo* purine biosynthesis and upregulate the *de novo* purine biosynthesis pathway, mTORC1 activates ATF4, boosts the translation of MTHFD2, and increases the expression of serine synthesis and other enzymes in the mTHF cycle (36). MTHFD2 expression regulates how much formates are released from the mitochondria into the cytoplasm. Tetrahydrofolate is finally incorporated by formate via methylenetetrahydrofolate dehydrogenase 1 (MTHFD1) once they are in the cytosol, producing the 10-formyltetrahydrofolate cofactors necessary for GART and ATIC activity and subsequent IMP synthesis (37). See **Figure 1**.

Purinosomes are formed in cells as a result of mitochondrial dysregulation, and the reduction of MTHFD2 transcription following mitochondrial DNA depletion leads to a decrease in serine processed in one-carbon metabolism. The study found that FGAMS and ADSL in purinosome composition enzymes co-isolate from mitochondria in a rapamycin-dependent manner, implying that inhibiting mTOR disrupts purinosome-mitochondrial colocalization and inhibits purinosome formation stimulated by mitochondrial dysregulation (19).

mTOR has been demonstrated to stimulate the expression of genes involved in the pentose phosphate pathway, which results in the manufacture of PRPP, the substrate for the first step in the purine biosynthesis pathway (38–40). These findings imply that mTOR plays a critical regulatory role in the *de novo* purine biosynthesis pathway.

### HIF-1 signaling pathway

Purinosome plays a role in tumor metabolism regulation by influencing the hypoxia inducible factor 1 (HIF-1) signaling pathway. As previously stated, tumor cells exhibit the Warburg effect, and glycolysis is active, with one of the causes being the activation of hypoxic-inducing molecules (41). Purinosome buildup in low oxygen environments can stabilize HIF-1α subunits and promote HIF-1 activation. HIF-1 can influence tumor cell metabolic adaptability and enhance glycolysis and lactic acid generation.

It has been discovered that cells have enhanced purinosome assembly when exposed to hypoxia. A class of proteins called hypoxia-inducible factors regulates how cells react to low oxygen levels and is essential for the construction of purinosome complexes under hypoxia. In cells lacking ATIC, hypoxia-driven purinosome assembly is suppressed. The glycolytic flux shifts into the pentose phosphate and serine biosynthesis pathways to provide enough PRPP and serine to maintain purine production, not the upregulation of transcription or translation of the enzymes within the *de novo* purine biosynthesis pathway, which would otherwise be the cause of the increase in purinosome formation under hypoxia (42).

## Prospects

Only a small portion of the purine that the human body needs to synthesize on its own comes from diet. The primary purine synthesis mechanism *in vivo* is *de novo* purine biosynthesis. To keep the purine pool homeostasis in organisms, this process works in coordination with the rescue synthesis pathway of purine nucleotides. *De novo* purine synthetase builds purinosomes, which are structures that are focused on transferring and processing substrates quickly along the enzymatic pathway. Purinosomes are important because they boost intracellular metabolic flow effectiveness when there is a strong demand for purines. Purinosomes have been used as a therapeutic target as research has progressed, and signaling mechanisms connected to purinosome synthesis and breakdown, such as the mTOR signaling pathway, have been gradually identified. However, as current studies focus more on the formation conditions and components of purinosomes, the reference content of this paper is limited.

Due to its important role in tumor development, purinosome has become a potential target for cancer therapy. Anticancer medicines that target the *de novo* purine biosynthesis pathway are now classified into three types. For starters, purine analogues like 6-mercaptopurine are structurally identical to inoxanthine, which interferes with purine nucleotide synthesis and prevents cell development. The second type of analogue is amino acid analogues, such as aza-serine, which are structurally similar to glutamine and can disrupt the reaction in which glutamine participates in purine nucleotide synthesis. Third, folate analogues, such as methotrexate, down-regulate purine nucleotide synthesis by competitively inhibiting dihydrofolate reductase and lowering the level of tetrahydrofolate, the substrate for purine nucleotide synthesis. Anticancer drugs targeting enzymes related to purine *de novo* synthesis pathway collected from DrugBank include fluorouracil, mercaptopurine, etc. See **Table 1** for details.

**Table 1:** Anticancer drugs that target enzymes involved in the purine de novo synthesis.

Generic Name	Targets	Diseases
Fluorouracil	PPAT	basal cell carcinoma, colorectal carcinoma (43)
Dasatinib	PPAT	Philadelphia chromosome-positive acute lymphoblastic leukemia (44)
Mercaptopurine	PPAT/IMPDH	acute lymphocytic leukemia (45)
Pemetrexed	GART	mesothelioma (46), colorectal cancer (47)
Alanosine	ADSS	brain cancer (48)

PPAT, phosphoribosyl pyrophosphate amidotransferase; IMPDH, inosine-5'-monophosphate dehydrogenase; GART, GARS-GAR transformylase-aminoimidazole ribonucleotide synthetase; ADSS, Adenylosuccinate synthetase.

The argument made in this article is that future cancer treatments will increasingly focus on purine metabolism and target the construction and disassembly of purinosomes. Drugs made for purinosomes are still being developed, despite the fact that there are numerous therapeutic targets for the *de novo* purine biosynthesis pathway. Further research and discussion are needed to determine whether medications targeting the purinosome, a multi-enzyme complex, can be targeted by medications targeting the *de novo* purine biosynthesis pathway. There may also be distinctions between medications targeting the two. Some medications thought to target the *de novo* purine biosynthesis pathway may not be able to enter the purinosome structure and may only be able to act prior to the purinosome’s formation. However, there should also be medications that prevent the synthesis of purinosomes and subsequently purine metabolism. These medications may act as substrates or enzyme analogues in the construction of purinosomes, which would then directly participate in the competitive inhibition of purine synthesis by inhibiting the compartmentalization of purinosomes. It’s also feasible that the pathway for *de novo* purine synthesis is disrupted by the direct suppression of purinosome assembly. These hypotheses pave the path for further research on targeted purinosome medicines. Of course, the fact that the purinosomes mentioned above rely on microtubule networks makes them a possible target for anticancer drug development. At the same time, the AMPK, mTOR, and HIF-1 signaling pathways must be given more consideration when developing anti-cancer medications that target purinosomes. It is anticipated that these medications will have a very promising future.

## Author contributions

CZ: Funding acquisition, Supervision, Writing – review & editing. JX: Investigation, Methodology, Resources, Software, Writing – original draft, Writing – review & editing. JL: Writing – review & editing. XC: Writing – review & editing.
